# Response of underwater photosynthesis to light, CO_2_, temperature, and submergence time of *Taxodium distichum*, a flood-tolerant tree

**DOI:** 10.3389/fpls.2024.1355729

**Published:** 2024-03-19

**Authors:** Jinbo Guo, Jianhui Xue, Yunlong Yin, Ole Pedersen, Jianfeng Hua

**Affiliations:** ^1^ Institute of Botany, Jiangsu Province and Chinese Academy of Sciences, Nanjing, China; ^2^ College of Biology and the Environment, Nanjing Forestry University, Nanjing, China; ^3^ Institute of Botany, Jiangsu Province and Chinese Academy of Sciences, Jiangsu Key Laboratory for the Research and Utilization of Plant Resources, Nanjing, China; ^4^ Department of Biology, University of Copenhagen, Copenhagen, Denmark; ^5^ School of Biological Sciences, The University of Western Australia, Crawley, WA, Australia

**Keywords:** bald cypress, contact angle, flood tolerance, gas films, hydrophobicity, swamp cypress, low oxygen quiescence syndrome

## Abstract

**Introduction:**

Partial or complete submergence of trees can occur in natural wetlands during times of high waters, but the submergence events have increased in severity and frequency over the past decades. *Taxodium distichum* is well-known for its waterlogging tolerance, but there are also numerous observations of this species becoming partially or complete submerged for longer periods of time. Consequently, the aims of the present study were to characterize underwater net photosynthesis (*P_N_*) and leaf anatomy of *T. distichum* with time of submergence.

**Methods:**

We completely submerged 6 months old seedling of *T. distichum* and diagnosed underwater (*P_N_*), hydrophobicity, gas film thickness, Chlorophyll concentration and needles anatomy at discrete time points during a 30-day submergence event. We also constructed response curves of underwater *P_N_* to CO_2_, light and temperature.

**Results:**

During the 30-day submergence period, no growth or formation new leaves were observed, and therefore *T. distichum* shows a quiescence response to submergence. The hydrophobicity of the needles declined during the submergence event resulting in complete loss of gas films. However, the Chlorophyll concentration of the needles also declined significantly, and it was there not possible to identify the main cause of the corresponding significant decline in underwater *P_N_*. Nevertheless, even after 30 days of complete submergence, the needles still retained some capacity for underwater photosynthesis under optimal light and CO_2_ conditions.

**Discussion:**

However, to fully understand the stunning submergence tolerance of *T. distichum*, we propose that future research concentrate on unravelling the finer details in needle anatomy and biochemistry as these changes occur during submergence.

## 1 Introduction

Partial or complete submergence of trees can occur in natural wetlands during times of high waters. Accordingly, more than 1,000 species of trees and bushes in Pantanal (one of the world’s largest tropical wetlands) become submerged every year in the wet season when the River Negro rises up to 10 m above its water level in the dry season (Parolin, 2009). However, trees can also face complete submergence in man-made wetlands such as at the banks of the Three Gorges Reservoir, where *Taxodium distichum* has been introduced in an attempt to stabilize the steep banks (Wang et al., 2019; Ding et al., 2021; He et al., 2021). Since the Three Gorges dam has first stored water in June 2003, plants growing at lower elevations, including the cultivated *T. distichum*, have experienced periodic complete submergence every year. In this case, *T. distichum* not only survived but grew from seedling to tree (Li et al., 2006). We propose that the outstanding flood tolerance of *T. distichum* is partly a result of its remarkable ability to photosynthesize under water, which slows down carbohydrate depletion and protects the tissue from anoxia via O_2_ production during extended periods of submergence.

In air, gases diffuse 10,000-fold faster than in water, and therefore, CO_2_ and O_2_ generally restrict photosynthesis and respiration of submerged terrestrial plants. Consequently, submerged aquatic plants have evolved a number of key shoot and root traits involved in facilitating CO_2_ or O_2_ exchange with the floodwater including, but not limited to, thin leaf lamina composed of only two cell layers, thin or completely absent leaf cuticle, chloroplasts in the leaf epidermis, and aerenchyma to facilitate internal aeration (Sculthorpe, 1967). However, even in the presence of these extreme adaptations, CO_2_ availability can still limit underwater photosynthesis (Madsen and Sand-Jensen, 1991; Maberly and Madsen, 2002), and about half of the world’s aquatic plant species have thus evolved the ability to use bicarbonate (HCO_3_^−^) as an alternative inorganic carbon source in photosynthesis (Prins and Elzenga, 1989; Iversen et al., 2019). Lacking most of these key leaf traits, the photosynthetic rates of submerged terrestrial plants are significantly lower than those of aquatic plants regardless of whether underwater photosynthesis is measured at ambient or elevated CO_2_ levels (Colmer et al., 2011). Similarly, the availability of molecular O_2_ can restrict underwater respiration of submerged terrestrial plants, and an O_2_ pressure of almost twice that of atmospheric equilibrium is needed to saturate respiration (Colmer and Pedersen, 2008). A recent meta-analysis encompassing 112 species of both aquatic and terrestrial plants have clearly demonstrated that partial or complete submergence lead to significant declines in tissue O_2_ status particularly during darkness when the only source of O_2_ for underwater respiration is O_2_ dissolved in the floodwater (Herzog et al., 2023). However, some species of wetland plants form numerous adventitious roots emerging from the stem and hanging into the floodwater as response to partial or complete submergence (Rich et al., 2013; Zhang et al., 2017; Lin et al., 2023). Such roots are referred to as aquatic adventitious roots and have been shown to act as “physical gills” by facilitating uptake of O_2_ from the floodwater (Ayi et al., 2016).

Some terrestrial plants, including the focal species of the present study, possess superhydrophobic leaves, and these have been shown to enhance gas exchange with the floodwater. Upon submergence, superhydrophobic leaves retain a thin gas film visible as a silvery sheen from the leaf surface (Pedersen and Colmer, 2012). Gas film formation on submerged leaves was first reported for deepwater rice, wheat, barley, and oats, where the beneficial effects on carbon fixation was also first reported (Raskin and Kende, 1983). Later, a series of studies reported leaf gas film formation during submergence in several species of wild wetland plants, where gas films were retained on partially or completely submerged leaves (Colmer and Pedersen, 2008; Pedersen et al., 2009; Winkel et al., 2016). The increased CO_2_ exchange caused by leaf gas films results in enhanced underwater net photosynthesis rate (*P_N_*), and generally underwater *P_N_* is 6- to 10-fold higher in the presence of leaf gas films compared with leaves without superhydrophobic leaves or with leaves where the gas films have been experimentally removed (Colmer and Pedersen, 2008; Pedersen et al., 2009; Winkel et al., 2011; Verboven et al., 2014; Konnerup et al., 2017; Winkel et al., 2017). Although the beneficial effects of leaf gas films have emphasized CO_2_ exchange for underwater photosynthesis, leaf gas films have also been shown to significantly enhance internal aeration. Accordingly, removal of hydrophobicity (and thereby also the leaf gas films) by brushing with a dilute detergent resulted in steep declines in O_2_ status of belowground tissues both in rice (Winkel et al., 2013) and in a wild wetland plant (Winkel et al., 2011), clearly demonstrating the crucial importance of leaf gas films for internal aeration of submerged terrestrial plants.

Low light availability under water may also restrict photosynthesis and submergence can invoke shade acclimation of submerged terrestrial leaves. In natural water bodies, the light intensity under water is lower than that in the air above not only because light is being reflected at the surface but also because light is being absorbed by water itself, by suspended particles such as algae, and by colored dissolved organic matter (Kirk, 1994). Murky floodwaters with algal blooms and/or high amounts of colored dissolved organic matter offer even less light for submerged plants with only 0.5% of the surface insolation left at deep floods (Vervuren et al., 2003). Consequently, many terrestrial plants respond to submergence by shade acclimations in their leaves, and these acclimations involve a reduction in leaf thickness, a thinner cuticle, thinner cell walls, and therefore a lower leaf mass area all resulting in better tissue O_2_ status (Mommer et al., 2007) and enhanced underwater *P_N_* due to better CO_2_ exchange (Mommer et al., 2004). Interestingly, the strong beneficial effect of leaf gas films on CO_2_ uptake is to a certain extent counteracted by the reflection of light at low light intensities; i.e., at low light, the silvery sheen of gas films reflects light and results in lower underwater *P_N_* (Winkel et al., 2017), showing that leaf gas films can also be disadvantageous during submergence in a low-light environment.

Trees and bushes forming the riparian vegetation often become partial or completely submerged when the river rises. However, poor flood tolerance of terrestrial plants leads to decreases in species richness as flooding intensity increases, leaving only the most flood-tolerant species to form the riparian vegetation (Garssen et al., 2017). Consequently, there are only two genera of Central European Trees showing very high flood tolerance (i.e., species of *Alnus* and *Salix*), and these are characterized with the formation of adventitious roots, lenticels, and aerenchyma in response to flooding (Glenz et al., 2006). However, the model species of the present study, *T. distichum* (L.) Rich, also shows extraordinary flood tolerance. It is a deciduous tree of the *Taxodium* genus, native to North America and Mexico where it forms large natural stands mostly in coastal plains affected by tide, in marshes with poor drainage, and in lowlands with periodic flooding (Wang et al., 2022; Guo et al., 2023). Owing to its outstanding flood tolerance, *T. distichum* has broad application prospects and is promoted for use in ecosystem restoration and construction of wetlands (Ding et al., 2021; He et al., 2021). More recently, it was found to have excellent tolerance to long-term periodic submergence in the water-level-fluctuating zone of the Three Gorges Reservoir (Wang et al., 2016, 2019). Interestingly, we found that *T. distichum* had a higher survival rate when submerged in winter than in summer, which may be because the activity of enzymes involved in underwater *P_N_* is affected by water temperature like other enzymes; thus, it is necessary to explore the response of underwater *P_N_* to temperature.

Consequently, the aims of the present study were to characterize underwater *P_N_* and leaf anatomy of *T. distichum* with time of submergence and to establish light, CO_2_, and temperature response curves of the underwater *P_N_*. Aerial photosynthesis of *T. distichum* has been thoroughly investigated (Wang et al., 2016; Taylor and Smith, 2017), but its capacity for underwater photosynthesis has not yet been evaluated. Interestingly, it was previously reported that *T. distichum* possesses a superhydrophobic leaf cuticle (Neinhuis and Barthlott, 1997), which should result in gas film formation during submergence. We therefore hypothesized that (i) some photosynthesis takes place when submerged, but the rate is strongly limited by light and CO_2_ showing a characteristic relationship with temperature; (ii) the underwater *P_N_* of *T. distichum* declines with time of submergence; (iii) and the decline in *P_N_* is linked to loss of leaf hydrophobicity and thereby the beneficial role of leaf gas films. Our study therefore fills an important gap related to the complete lack of knowledge related to the photosynthetic capacity of woody plants under water and the associated mechanistic understanding of flood tolerance of trees.

## 2 Materials and methods

### 2.1 Plant materials and growth conditions

Leaf material for characterization of key photosynthetic parameters was sampled from a 4-year old *T. distichum* (L.) Rich at the Institute of Botany, Jiangsu Province and Chinese Academy of Sciences (32°05’ N, 118°83 ‘E) during midmorning from 9 to 10 a.m. The plant height was 2.6 m high and the diameter at breast height was 2.32 cm, which was in a rapid growth phase. Young but fully expanded and healthy branchlets with green needle-like leaves of the linear-lanceolate type were chosen for these experiments in June–July, whereas scale-like and appressed needles were disregarded.

For the long-term submergence experiment, seeds of *T. distichum* were sown in a seedling tray and after germination transferred to pots (23 cm upper diameter, 15 cm basal diameter, and 22 cm high) filled with a mixture of potting soil and sandy clay. The seedlings were grown in a greenhouse (temperature: 23 ± 2°C; humidity: 60%–70%) for 2 months and then moved outdoors for another 4 months, and the pots were watered daily with tap water. 30 healthy seedlings with an average height of 45 cm were selected for the experiment.

### 2.2 CO_2_ versus underwater *P_N_*

Underwater *P_N_* was measured following the approach of Pedersen et al. (2013). In brief, artificial floodwater was prepared according to Smart and Barko (1985) with a final alkalinity of 2.0 mol H^+^ equivalent m^−3^. Considering that the underwater photosynthetic CO_2_ saturation concentration of submerged leaves of terrestrial wetland plants is approximately 20–75 times or even higher than the atmospheric equilibrium concentration (~18 mmol m^−3^ free CO_2_) (Pedersen et al., 2009), a CO_2_ concentration range of 10–2,000 mmol m^−3^ was set. In addition, compared with high CO_2_ concentration, underwater *P_N_* changes more significantly under low CO_2_ concentration; thus, six concentration gradients (10, 25, 50, 100, 200, and 500 mmol m^−3^) were set for low concentration and three concentration gradients (1,000, 1,500, and 2,000 mmol m^−3^) were set for high concentration. Prior to the pH adjustment, the solution was purged with N_2_ to reduce the O_2_ concentration approximately 30%–50% of air equilibrium to prevent photorespiration during incubation (Setter et al., 1989). The artificial floodwater was then siphoned into 44-mL glass vials and two pieces of 3-mm glass beads were added to each vial to ensure the mixing during incubation. One branchlet with needles (approximately 1 cm^2^ or approximately 15.8 mg fresh mass) before the vial was sealed with a glass lid (no headspace or gas bubbles present). The vials were mounted on a vertically rotating disk (10 rpm) and inundated in a constant temperature bath at 25°C and illuminated with a photon flux of 1,000 µmol m^−2^ s^−1^ (see below). Vials without tissue served as blanks.

After 60 min, the vials were retrieved and the O_2_ concentration was measured using an O_2_ optode (OPTO-MR, Unisense, Denmark) inserted into the vial. The needles in the vial were then neatly placed on a clean white background board while a ruler is placed to take the photo. Make sure the leaves do not overlap each other when taking the photo; ImageJ software was used to measure the exact leaf area (Schneider et al., 2012). The photosynthetic rate was calculated using the following equation:


$$P_{N}(\mu mol\;O_{2}\;m^{-2}s^{-1})=\frac{\Delta O_{2}(\mu mol\;O_{2}\;L^{-1})V_{vial}(L)}{t(sec)A(m^{2})}$$

where ΔO_2_ is the difference in O_2_ concentration in vial with tissue and blanks, *V*_vial_ is the volume of the vials, i.e., 0.044 L, *t* is the incubation time, and *A* is the area of the needles.

### 2.3 Light versus underwater *P_N_*

Floodwater and tissue were prepared as above but with a fixed CO_2_ concentration of 500 mmol m^−3^. Compared with high light intensity, underwater *P_N_* changes more significantly under low light intensity; thus, six gradients (0, 50, 100, 200, 300, and 500 µmol photons m^−2^ s^−1^) were set for low light intensity and three gradients (1,000, 1,500, and 2,000 µmol photons m^−2^ s^−1^) were set for high light intensity. Different light intensities were established by using a high-pressure Na lamp at various distances and by regulating the voltage. For zero light, the vials were wrapped in aluminum foil. The light intensities were measured using a spherical PAR (photosynthetically active radiation) sensor (QSL2101, Biospherical Instruments Inc., USA). As for the CO_2_ response (see above), samples were incubated for 60 min.

### 2.4 Temperature versus underwater *P_N_*

Floodwater and tissue were prepared as above but with a fixed CO_2_ concentration of 500 mmol m^−3^ and PAR at 1,000 µmol photons m^−2^ s^−1^. The temperature was set to 10, 15, 20, 25, 30, or 35°C using a combination of an immersion heater (300W, SUNSUN, Zhejiang, China) and a water cooler (TECO, Taiwan, China) to achieve a stable temperature, which was monitored in real time by a temperature electrode (Temp-UniAmp thermosensor, Unisense, Denmark). As for the CO_2_ response (see above), samples were incubated for 60 min.

### 2.5 Long-term submergence

A 30-day submergence experiment with two treatments (drained controls or completely submerged) was conducted using 6-month-old *T. distichum* seedlings. Fifteen pots with one plant in each were transferred to three plastic tanks (depth, 69 cm; volume, 122 L) filled with tap water, with five seedlings (technical replicates) in each tank (true replicates). The water was changed every 2 days, and the hose was placed at the bottom of the bucket to ensure that the water was completely replaced. Another 15 plants served as controls, and these were unsubmerged and kept under conditions (photoperiod and temperature) similar to those for submerged plants and irrigated every 2 days. Underwater *P_N_* was measured on healthy needles on days 7, 14, 21, and 31 using a PAR of 1,000 µmol photons m^−2^ s^−1^ and a CO_2_ concentration of 500 mmol m^−3^ (see experimental procedure above) and leaves were transported in water back to the laboratory to minimize damage and exposure to air. It should be noted that the underwater *P_N_* of the submerged leaves of terrestrial wetland plants will be severely limited by the CO_2_ availability at atmospheric equilibrium CO_2_ concentrations. In order to more accurately evaluate the underwater photosynthetic capacity of submerged leaves, a CO_2_ concentration higher than atmospheric equilibrium was required, and 500 mmol m^−3^ is set for this experiment.

During the first 8 days of the submergence experiment, submerged leaves were harvested every other day. These were observed and photographed with scanning electron microscopy (SU8100, Hitachi Scientific Instruments, Japan). Subsequently, stomatal density (number of stomata per mm^−2^) and stomatal index (ratio of number of stomata to the total number of epidermal cells including stomata) were calculated from these images (Hegde and Krishnaswamy, 2021; Li et al., 2022).

At the end, healthy leaves were sampled and then transported into water to minimize damage and re-exposure to air. Chlorophyll measurements were conducted on submerged leaves as well as controls using ethanol extractions and absorbance of the extract was measured on a spectrophotometer (UV-1800, Shanghai Mepuda Instrument Co., LTD, China). Chlorophyll was calculated using the equations in Zhang et al. (2020).

Finally, cross-sections for microscopy were prepared from paraffin-embedded needles and later studied using visible light microscopy (BX53F, Olympus, Tokyo, Japan).

### 2.6 Influence leaf gas films on underwater *P_N_*

To investigate the effects of leaf gas films on underwater *P_N_*, four healthy 6-month-old *T. distichum* seedlings were selected. Two fully unfolded branchlets were sampled from each plant and then they were divided into two groups. To remove hydrophobicity and thereby prevent formation of leaf gas films, one group was brushed five times, on both sides, with a fine paintbrush dipped into 0.01% (v/v) Triton X. After that, they were washed for 5 s, three times, in artificial floodwater without Triton X (Teakle et al., 2014; Winkel et al., 2014). The other group was untreated and served as control with each group having four replicates. Underwater *P_N_* was measured as described above with 500 mmol CO_2_ m^−3^ under a photon flux of 1,000 µmol photons m^−2^ s^−1^ at 25°C.

### 2.7 Assessment of gas film thickness and needle hydrophobicity

Leaf gas film thickness was measured following the approach of Raskin and Kende (1983). In brief, the buoyancy of a branchlet was measured on five replicates using a four-digit balance with a hook underneath before and after removal of hydrophobicity using 0.01% Triton X; see above. Next, the area of the needles was determined (see above), and the gas film thickness (m) was calculated as gas film volume (m^3^) divided by needle area (m^2^).

Surface hydrophobicity was assessed by measuring the contact angle of a 1-mm^3^ droplet of water on the needle surfaces following Sikorska et al. (2017). Branchlets with needles were held horizontal using a glue stick. Water droplets were applied to the lamina of 10 replicate needles (5 on the adaxial side and 5 on the abaxial side), and photographed at ×35 magnification using a horizontally positioned dissecting microscope (MZ62, Mshot, China) and a digital camera. The droplet contact angles were measured using ImageJ (ImageJ v.1.43U, National Institutes of Health, Bethesda, MD, USA).

Finally, specific leaf area (SLA) was measured by determining the area (see above) and dry mass of needles, where the needles were dried for 48 h at 60°C. SLA was calculated as area (m^2^) divided by dry mass (kg).

### 2.8 Data analysis

We used non-linear regression to fit models derived from FvCB (CO_2_ response) (Liang and Liu, 2017) and light response was fitted according to the Ye model (Ye et al., 2013). The temperature optimum was modeled using a Gaussian function and a standard exponential function was used to predict temperature coefficient *Q*_10_ (Pedersen et al., 2016). The temperature coefficient *Q*_10_ represents the relative change of underwater *P_N_* with every 10°C change in temperature. The data were processed using Excel 2016 and graphed with Origin software (2021 64Bit, Electronic Arts Games, USA). Results were expressed as means ± standard deviation. All statistical tests were conducted using SPSS 16.0s (SPSS Inc., USA) including that of the Duncan’s multiple range test. Unless otherwise stated, a probability level of 0.05 was used.

## 3 Results

### 3.1 Submergence of *Taxodium* during times of high water level

**Figure 1:**
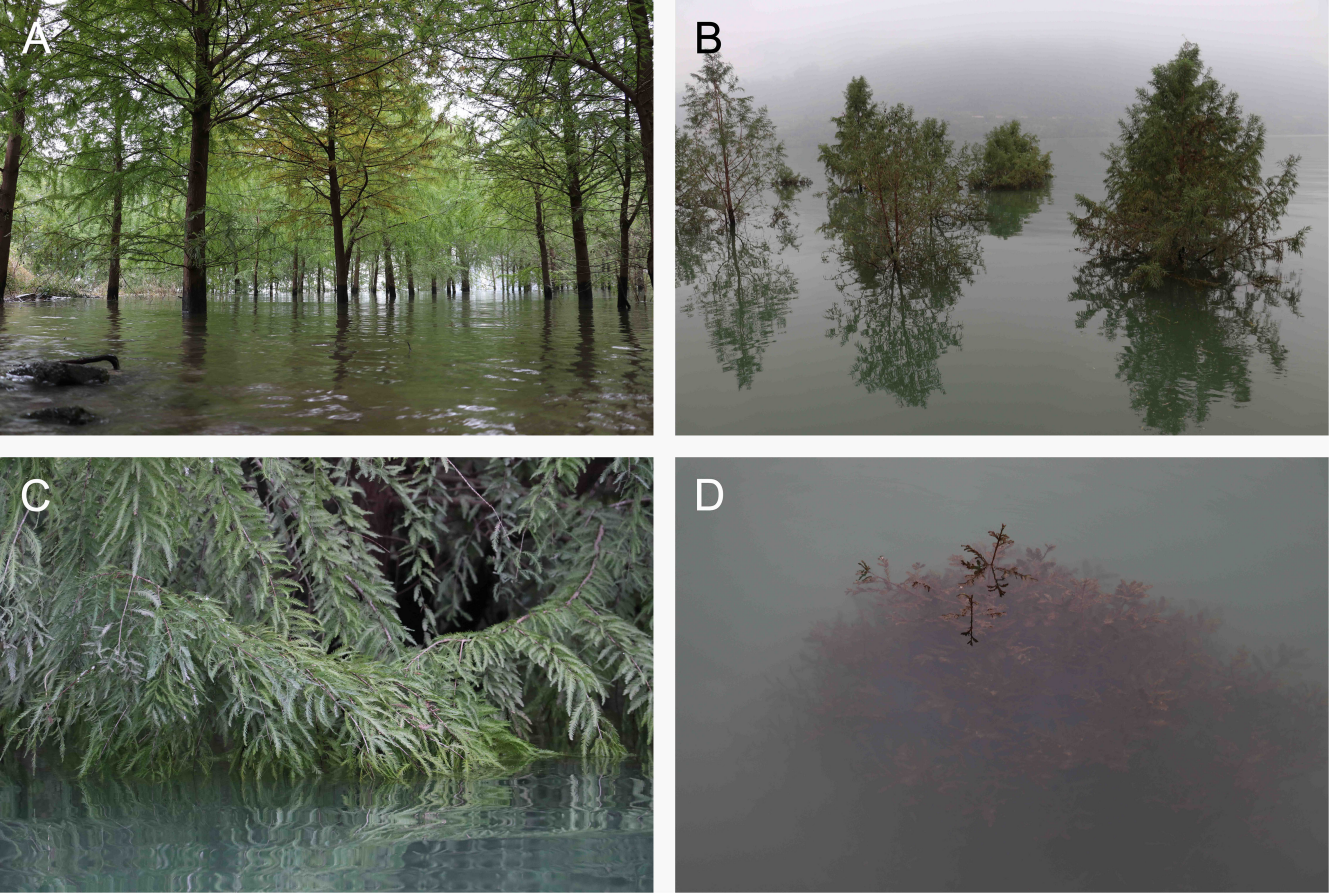
Habitat photos of *Taxodium* growing along the banks of the Three Gorges Reservoir, which is part of the Yangtze River. The species depicted is hybrid between *Taxodium distichum* and *Taxodium mucronatum*, which has been planted on the banks in an attempt to reduce erosion as the annual water level fluctuations are up to 175 m (Wang et al., 2019). **(A)** The initial phase of flooding resulting in waterlogging, but as the water continues to rise, the low branches become submerged **(B, C)**. Finally, the entire canopy is under water **(D)** and may remain so for up to 120 days and still survive (Yang et al., 2023).

Waterlogging, partial submergence, and even complete submergence of *Taxodium* is a recurrent phenomenon during times of high water levels. When the water first starts rising, the soil becomes flooded, resulting in waterlogging of vast areas of trees (**Figure 1A**), and as the water continues to rise, the lower branches become submerged (**Figures 1B, C**). Ultimately, the entire canopy is under water (**Figure 1D**), and gas exchange with the atmosphere is no longer possible. The floodwater in the Yangtze River is murky as a result of suspended materials (**Figure 1D**), and therefore the light environment also changes upon submergence, resulting in reduced photosynthesis due to the combination of low light and restricted CO_2_ availability. The habitat photos in **Figure 1** clearly demonstrate the relevance of our study, where we are aiming to characterize the ability of *T. distichum* to continue photosynthesizing under water with emphasis on response to CO_2_ and light availabilities, and temperature.

### 3.2 Response of underwater net photosynthesis to CO_2_, light, and temperature by *Taxodium distichum*

**Figure 2:**
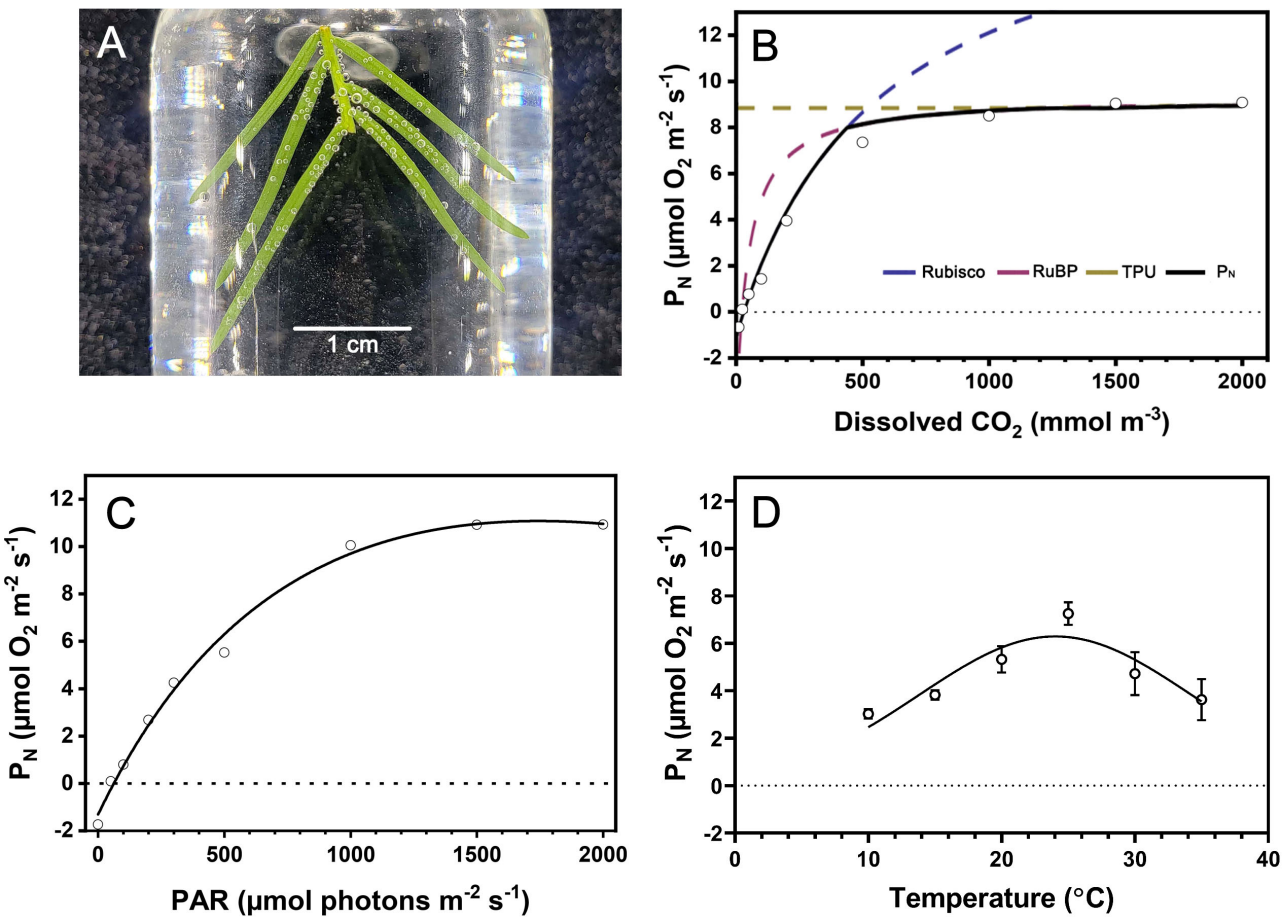
Response of underwater photosynthesis to CO_2_, light, and temperature of submerged *Taxodium distichum* branchlets. In **(A)**, a section of a branchlet is incubated in artificial floodwater in a glass vial, and the gas bubbles forming on the leaves show that O_2_ is being produced in underwater photosynthesis. **(B)** Underwater net photosynthesis (*P_N_*) at PAR = 1,000 µmol photons m^−2^ s^−1^ as a response to CO_2_ dissolved in the floodwater followed a saturation curve and is fitted to the means using the FvCB model (*R* ^2 ^= 0.99). **(C)** Similarly, underwater *P_N_* was fitted to a general light response curve (Ye model, *R* ^2 ^= 0.997) measured with 500 µmol L^−1^ dissolved CO_2_ in the floodwater to enable estimation of *P_N_*_max_ (10.97 µmol O_2_ m^−2^ s^−1^). In **(D)**, the response of *P_N_* to temperature is shown with 500 µmol L^−1^ dissolved CO_2_ in the floodwater and PAR = 1,000 µmol photons m^−2^ s^−1^, and using a Gaussian fit revealed an optimum for *P_N_* at 25°C. Data points in B–D show the mean ± SD (*n* = 4).

We characterized the response of underwater net photosynthesis (*P_N_*) to dissolved CO_2_, light availability, and temperature, which are relevant environmental parameters during submergence of *T. distichum*. Submerged branchlets with needles produced O_2_ when incubated in the artificial floodwater in the light visible as bubble formation on the needles (**Figure 2A**). The response of *P_N_* (i.e., net O_2_ consumption) to dissolved CO_2_ at 25°C and a photon flux of 1,000 µmol photons m^−2^ s^−1^ showed a typical saturation response, increasing as CO_2_ was raised to 2,000 mmol m^−3^ (**Figure 2B**). The FvCB model estimated the maximum carboxylation rate (*V*_cmax_), maximum electron transfer rate (*J*_max_), and day respiratory rate (*R*_day_) to be 19.73 µmol m^−2^ s^−1^, 39.34 µmol m^−2^ s^−1^, and 0.3 µmol m^−2^ s^−1^, respectively. However, the CO_2_ compensation point (Γ*) and CO_2_ saturation point (*C*_i,TUP_) are 37.43 mmol m^−3^ and 2,334 mmol m^−3^, which are approximately 2-fold and 130-fold atmospheric equilibrium (~18 mmol m^−3^ free CO_2_), respectively (**Table 1**).

**Table 1:** *Taxodium distichum* response curve parameter estimation and goodness of fit.

Parameters	Estimated value
*P_N_*–CO_2_ response curve (FvCB model)	
* V*_cmax_	19.73 µmol m^−2^ s^−1^
* J*_max_	39.34 µmol m^−2^ s^−1^
* R*_day_	0.30 µmol m^−2^ s^−1^
* C*_i,TUP_	2334 mmol m^−3^
Γ*	37.43 mmol m^−3^
* R* ^2^	0.99025
*P_N_*–light response curve (Ye model)	
α	0.0221
* P_N_*_max_	10.97 µmol m^−2^ s^−1^
* R*_dark_	1.31 µmol m^−2^ s^−1^
* I*_m_	1666.67 µmol m^−2^ s^−1^
* I*_c_	65.38 µmol m^−2^ s^−1^
* R* ^2^	0.997

V_cmax_, maximum carboxylation rate; J_max_, maximum electron transfer rate; R_day_, daily respiration rate; C_i,TUP_, CO_2_ saturation point; Γ*, CO_2_ compensation point; R^2^, coefficient of determination; α, initial quantum efficiency; P_N max_, maximum net photosynthetic rate at light saturation; R_dark_, dark respiration rate; I_m_, light saturation point; I_c_, light compensation point.

We diagnosed *P_N_* at contrasting light availabilities with 500 µmol CO_2_ L^−1^ in the floodwater. As expected, underwater *P_N_* also followed a saturation response with increasing light availability. In darkness, the dark respiration (*R*_dark_) of branchlets with needles was 1.31 µmol O_2_ m^−2^ s^−1^ (**Figure 2C**). Using the equation from Ye et al. (2013), the light compensation point (*I*_c_) and light saturation point (*I*_m_) were 65.38 µmol photons m^−2^ s^−1^ and 1,666.67 µmol photons m^−2^ s^−1^, respectively. *P_N_*_max_ was estimated to 10.97 µmol O_2_ m^−2^ s^−1^ at the given environmental conditions, i.e., dissolved CO_2_ at 500 µmol L^−1^ at 25°C (**Table 1**).

Underwater *P_N_* followed an exponential increase with increasing temperature in the tested interval from 10 to 25°C whereafter it steeply decreased with increasing temperature. Using a Gaussian model, we estimated the temperature optimum for underwater *P_N_* in *T. distichum* to 25°C with 500 µmol L^−1^ dissolved CO_2_ in the floodwater at a photon flux of 1,000 µmol photons m^−2^ s^−1^ (**Figure 2D**). Using the same dataset, but without considering temperatures exceeding the optimum for underwater *P_N_*, we estimated the *Q*_10_ of *P_N_* to 1.84 demonstrating the strong dependence of underwater *P_N_* on environmental temperature.

### 3.3 Hydrophobicity and gas film retention by needles of *Taxodium distichum*

**Figure 3:**
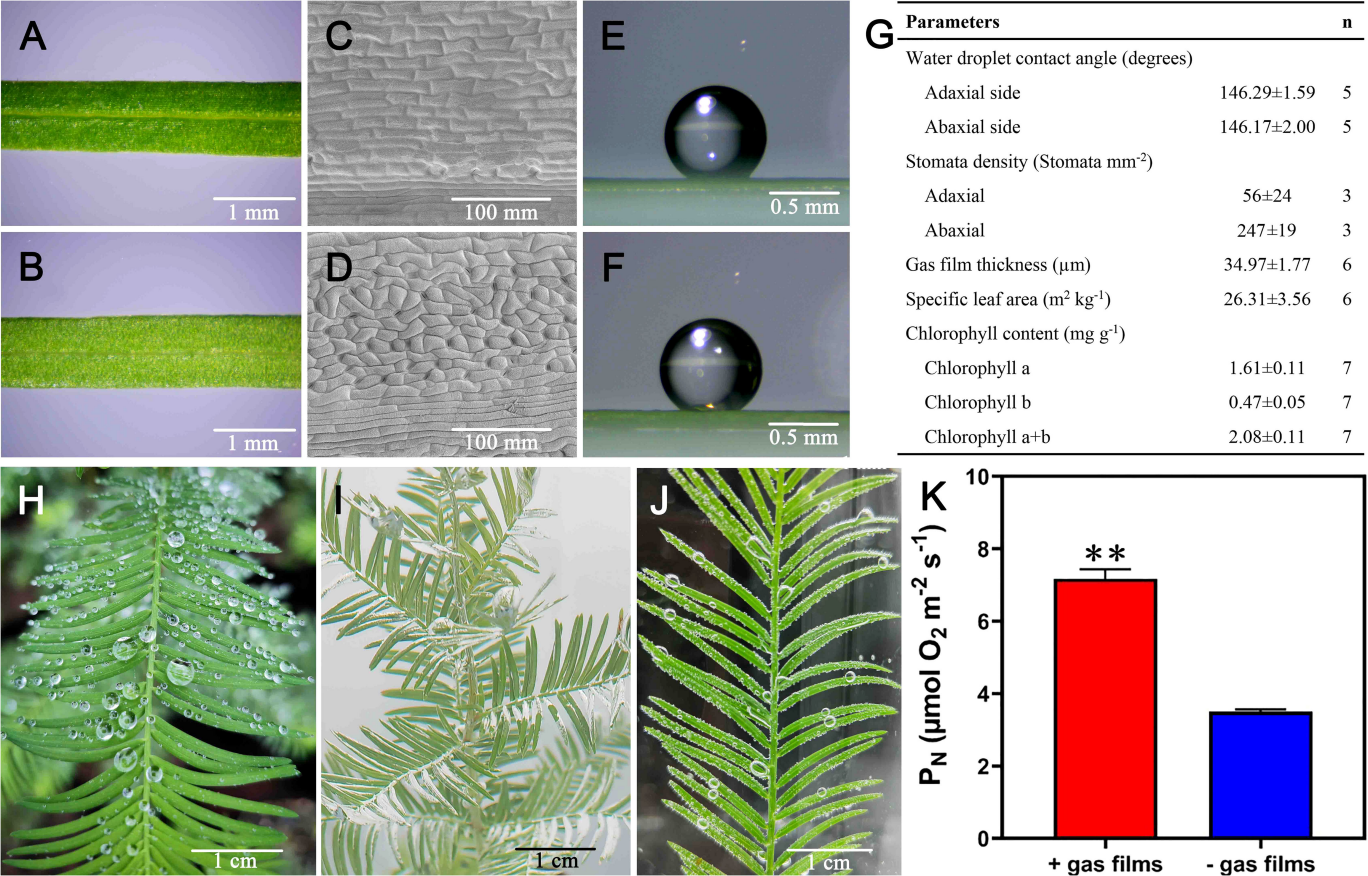
Adaxial **(A, C, E)** or abaxial **(B, D, F)** view of the needle surface close to the mid-vein, scanning electron micrograph of the cuticle and lateral view of a 1 mL water droplet. More details are shown in **(G)**. The needles in the air repel water **(H)**, and retain a thin gas film upon submergence visible as a silvery sheen **(I)**. When submerged in light, underwater photosynthesis results in bubble formation on the needle surfaces **(J, K)** shows the effect of gas film on underwater net photosynthesis measured at PAR = 1,000 µmol photons m^-2^ s^-1^ and CO_2_ at 500 µmol L^-1^. In **(K)**, ** indicates P < 0.01, one-tailed Student’s t-test.

At the onset of submergence, *T. distichum* forms a thin gas film on its needles and therefore we aimed at characterizing hydrophobicity, gas film thickness, and other key features known to influence underwater *P_N_*. Macroscopically, the needles are very similar on their adaxial and abaxial sides, but stomatal density differs with more than fourfold higher density of stomata on the abaxial side (**Figures 3A–D, G**). However, the water-repellent traits were similar, showing a contact angle of 146° on both sides, and although these angles only render the needles hydrophobic (and not superhydrophobic) (Koch and Barthlott, 2009), the hydrophobicity was nevertheless sufficient to initially retain a 35-µm-thick gas layer upon submergence (**Figures 3E–G**). The needles of *T. distichum* in air have been observed to repel water, and the gas film formed underwater is directly visible as a silvery sheen (**Figures 3H, I**), and its well-described facilitation of underwater photosynthesis is evident from the bubble formation when the branchlets are submerged in CO_2_-rich water in the light (**Figure 3J**). We also manipulated needle hydrophobicity to enable a direct comparison of underwater *P_N_* of needles with or without a gas film, and we found that the gas film increased underwater *P_N_* 2.1-fold as compared with needles where gas film formation was prevented (**Figure 3K**).

### 3.4 Response of *Taxodium distichum* to long-term submergence

**Figure 4:**
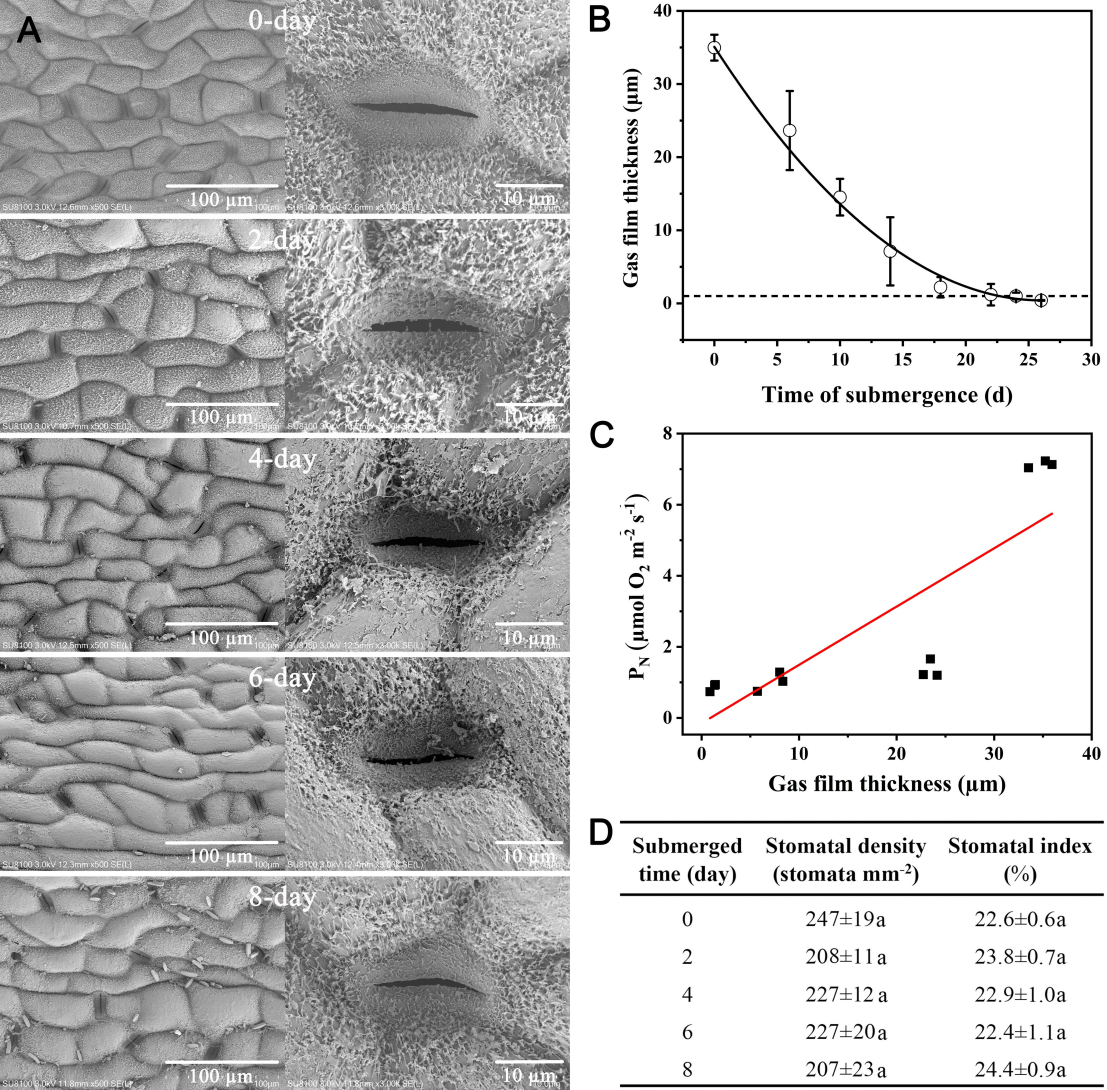
Changes in needle surface structure, gas film thickness, and underwater *P_N_* with time of submergence of *Taxodium distichum*. Scanning electron micrographs **(A)** show the changes in surface structure immediately before submergence (day 0, control) and days 2, 4, 6, and 8. Decline in gas film thickness **(B)** during the 30 days of submergence and the relationship between gas film thickness and underwater *P_N_* **(C)**. A Pearson correlation analysis showed a correlation coefficient of 0.83 and *p*< 0.01. The table **(D)** shows stomatal aperture, density, opening rate and index, and the same time points. Data are means ± SD, *n* = 3–5. Different letters within the same column of data indicate *p*< 0.05 (Duncan’s multiple comparison).

The fact that *Taxodium* can become completely submerged for several months prompted us to conduct a controlled laboratory experiment where we submerged 6-month-old plants for 30 days. During the first 24 days of submergence, the hydrophobicity was lost and the leaf cuticle gradually became colonized with bacteria with the first bacterial cells appearing already after 2 days of submergence (**Figure 4A**). The colonization of bacteria was accompanied by a decline in hydrophobicity, and leaf gas films dramatically decreased during the first few days of submergence (**Figure 4B**). This loss of gas films resulted in a significant decline in underwater *P_N_* as indicated by the significant positive correlation between gas film thickness and under *P_N_* (**Figure 4C**). Key stomatal features (stomatal density and stomatal index) did not change during the first 8 days of submergence (**Figure 4D**).

**Figure 5:**
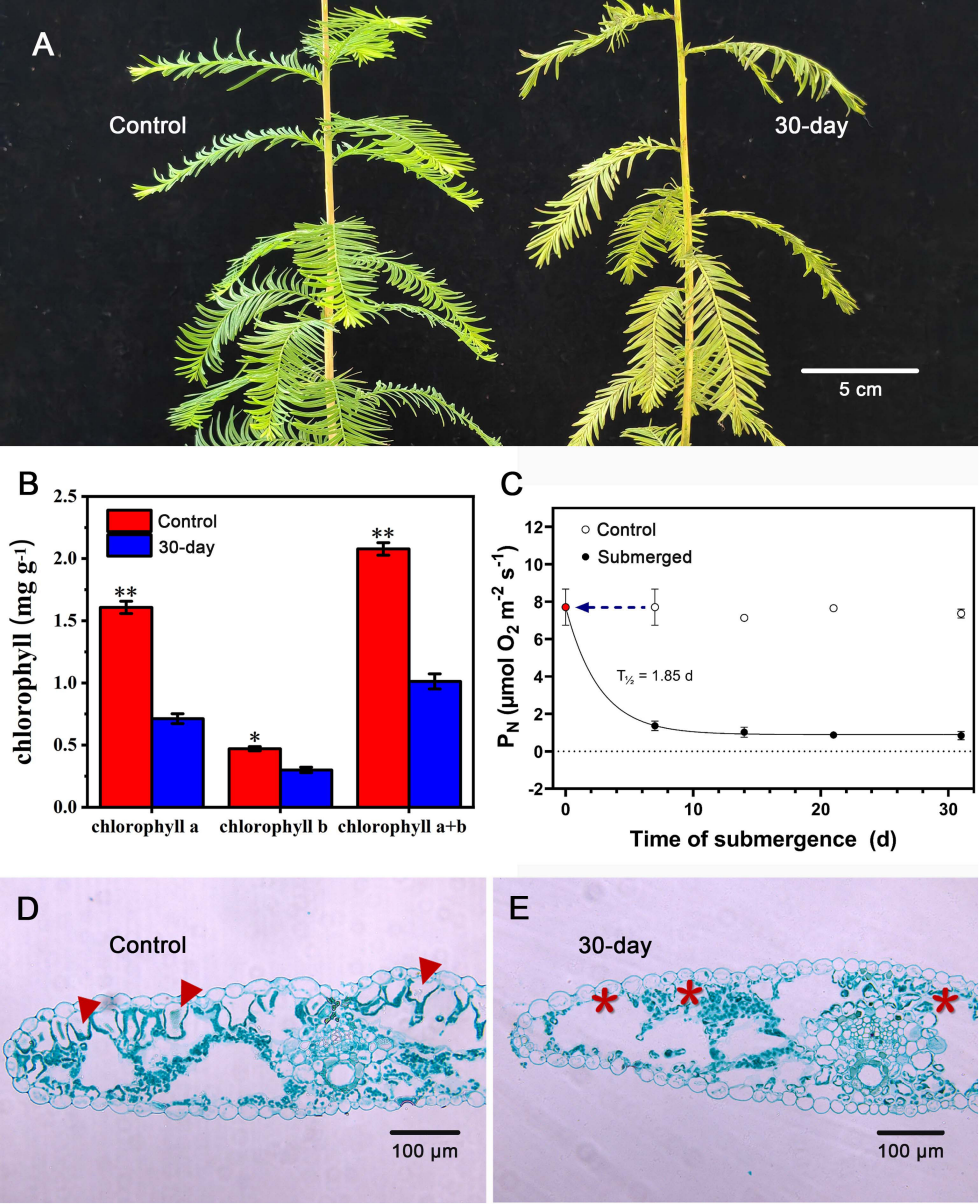
Response to long-term submergence by *Taxodium distichum*. **(A)** shows habitus photos of an unsubmerged control plant and a plant that has been completely submerged for 30 days. In **(B)**, chlorophylls (Chl_a_, Chl_b_, and Chl_a+b_) are shown for unsubmerged control needles and needles that have been submerged for 30 days. **(C)** shows underwater net photosynthesis (*P_N_*) with time of submergence along with control measurements on unsubmerged branchlets at each sampling point. Data are means ± SD (*n* = 4), and * and ** indicate *p*< 0.05 and *p*< 0.01, respectively (one-tailed Student’s *t*-test), and the half-life of *P_N_* was calculated using an exponential decay function. Below, **(D, E)** show cross-sections of an unsubmerged needle and a needle that has been submerged for 30 days. To the left, arrowheads point at palisade tissues, and to the right, * indicates missing palisade tissues.

However, over the entire submergence period of 30 days, significant changes took place at the needle level. The needles started yellowing (**Figure 5A**), and the yellow was also reflected in a significant decline in chlorophylls (**Figure 5B**). The combined effect of loss of hydrophobicity and the decline in chlorophylls resulted in a steep decline in underwater photosynthesis already within the first 8 days of submergence with a predicted *T*_½_ in *P_N_* of 1.85 days (**Figure 5C**). In addition to the biochemical changes in chlorophyll concentration, the needles also underwent anatomical changes during the 30 days of submergence as all of the palisade cells degraded (**Figures 5D, E**). Importantly, even with the loss of palisade tissues and the significant declines in chlorophylls, the needles maintained some capacity for underwater *P_N_* during the entire submergence period as underwater *P_N_* never declined below 1 µmol O_2_ m^−2^ s^−1^.

## 4 Discussion

Partial or complete submergence of trees is a common phenomenon in several natural or man-made wetlands (Parolin, 2009) and yet the ability of the leaves to photosynthesize under water had not yet previously been studied. In the present study, we found that the needles of *T. distichum* are hydrophobic and retain a thin gas film during submergence, and the gas films greatly enhanced underwater *P_N_* through their beneficial effect on gas exchange between needles and floodwater. We also found that the needle hydrophobicity was lost with time of submergence, but even after a month of complete submergence, the needles still maintained some capacity for underwater *P_N_*. Nevertheless, the needles had undergone structural and biochemical changes with loss of mesophyll cells and significant declines in chlorophyll concentrations. Below, we are discussing these findings in the context of existing knowledge on flood tolerance of terrestrial plants with emphasis on beneficial leaf traits such as leaf hydrophobicity, gas film formation, and SLA, and we also identify areas of exploration to fill in the many knowledge gaps that still remain.

### 4.1 Hydrophobicity of *Taxodium distichum* needles and gas film formation

Terrestrial leaves generally perform poorly under water due to the restricted gas exchange in water compared with air resulting in restricted O_2_ uptake for respiration or CO_2_ uptake for photosynthesis. The poor performance has been clearly demonstrated using the model plant, *Rumex palustris*, showing that underwater *P_N_* of aerial leaves was only 0.5% of the rate in air (Mommer et al., 2006). In stark contrast, leaves of *R. palustris* formed under water could attain rates of underwater *P_N_* at 35% of that in air showing the great benefit of leaf acclimation to underwater gas exchange. However, leaf acclimation is only a feasible strategy for long-term submergence, as production of new aquatic leaves requires reallocation of carbohydrates to fuel leaf. Instead, superhydrophobic leaves that retain a gas film under water have been shown to be a very competitive solution to enhance gas exchange without further investment in leaf acclimation (Colmer and Pedersen, 2008).

Hydrophobicity can be characterized using the contact angle of a microscopic water droplet. Accordingly, leaf cuticles with contact angles exceeding 150° are classified as superhydrophobic (with rice being a typical example) (Kwon et al., 2014), whereas those with contact angles less than 150°—but larger than 90° (Koch and Barthlott, 2009)—are hydrophobic. In the case of *T. distichum*, the contact angles were just below the 150° cut (146°, **Figure 3G**), but the needles nevertheless retained a gas film upon submergence (**Figure 3I**). The gas films forming on the needles of *T. distichum* were of similar thickness (35 µm, **Figure 3G**) to those formed by rice leaves (30–60 µm) (Winkel et al., 2014; Kurokawa et al., 2018; Mori et al., 2019) or by wheat leaves (20–40 µm) (Konnerup et al., 2017). Consequently, it is not surprising that the beneficial effects on gas exchange and underwater *P_N_* were significant.

In water, gas diffusion is slow and therefore physiological processes relaying on gas exchange can become restricted by slow substrate supply such as CO_2_ for photosynthesis. However, leaf gas films greatly enhance gas exchange (Verboven et al., 2014), and we found that needles with gas films achieved twofold higher photosynthetic rates compared with needles that had the hydrophobicity removed and where gas films therefore did not form (**Figure 3K**). It has previously been found that leaf gas films can increase underwater *P_N_* up to three- to sevenfold (Colmer and Pedersen, 2008), but the photosynthesis in these experiments was assessed at lower (200 µM) external CO_2_ concentrations, where the beneficial effect of gas films on gas exchange is more pronounced (Winkel et al., 2017). Interestingly, the underwater *P_N_* obtained at saturating light and CO_2_ levels in the present study matched those of *P_N_* in air; i.e., in both environments, the rates were approximately 10–12 µmol O_2_ m^−2^ s^−1^ (**Figures 2B, C**) (Neufeld, 1983; Mommer et al., 2006), underlining the significant effect of gas films on the needles of *T. distichum*. Consequently, we propose that a key reason for the stunning flood tolerance of *T. distichum* is its ability to maintain a substantial photosynthetic activity during submergence resulting in both carbohydrate and O_2_ production. However, the realized photosynthetic rates greatly depend on both light and CO_2_ availability under water and, to a large extent, temperature as well.

### 4.2 Influence of CO_2_, light, and temperature on underwater *P_N_* of *Taxodium distichum*

CO_2_ uptake by *T. distichum* followed a classical FvCB response curve. However, underwater *P_N_* remained negative until the CO_2_ compensation point at 37.43 mmol m^−3^ was reached; below, the needles of *T. distichum* consumed more O_2_ than they produced. The CO_2_ compensation point is equivalent to approximately twofold that of atmospheric equilibrium (~18 mmol m^−3^ free CO_2_), demonstrating the importance of net CO_2_ production in the floodwater in order for the underwater *P_N_* to become positive and therefore result in significant carbohydrate production. The *C*_i,TUP_ (CO_2_ saturation point) was estimated to 2,334 mmol m^−3^, or 130-fold atmospheric equilibrium, which is higher than that of submerged rice (Winkel et al., 2013) and submerged wheat (Winkel et al., 2017), and even higher than that of submerged *Hordeum marinum* (Pedersen et al., 2010). It has been demonstrated that the underwater *P_N_* capacity of submerged plants was severely limited at atmospheric equilibrium CO_2_ concentrations (Pedersen et al., 2009). Although some studies found that the CO_2_ concentration recorded in flooded rice fields was 20–180 times the atmospheric equilibrium concentration (360–3,240 mmol m^−3^) (Setter et al., 1987), the diffusion rate of the gas in water is 10,000 times lower than in air, resulting in underwater *P_N_* being still limited (Pedersen et al., 2013). CO_2_ availability limitations are a long-standing challenge for submerged plants, but interestingly, rice, wheat, and *H. marinum* retain leaf gas films when submerged, which has been shown to significantly enhance gas exchange. The overall similarity of the CO_2_ response in *T. distichum* to the other three terrestrial species with leaf gas films is likely due to the physical effect of the gas films facilitating the exchange between needles and floodwater rather than physiological similarities among these distantly related species.

Light utilization by *T. distichum* under water also showed a saturating response to light when assessed with 500 µmol CO_2_ L^−1^ in the floodwater. As for CO_2_, underwater *P_N_* was initially negative at low light levels and only reached positive values (*I*_c_) at PAR > 65.38 µmol photons m^−2^ s^−1^ (**Figure 2C**). When PAR is higher than the saturation point (*I*_m_) of 1,666.67 µmol photons m^−2^ s^−1^, underwater *P_N_* reaches a maximum value (*P_N_*_max_) of 10.97 µmol m^−2^ s^−1^, which is very difficult to achieve due to light loss; thus, underwater *P_N_* is generally limited by PAR. The initial quantum efficiency (α) of 0.0221 absorbed was in the same order of magnitude as that of submerged wheat (Winkel et al., 2017) and *Phalaris arundinacea* (a terrestrial wetland species also forming leaf gas films upon submergence) (Vervuren et al., 1999; Winkel et al., 2017), whereas two other species without gas films utilized light much less efficiently (*Rumex crispus* and *Arrhenatherum elatius*) (Vervuren et al., 1999). These findings emphasize the importance of gas films also for light use efficiency as the light use relies not only on incident light reaching the leaf surfaces but also on entry of CO_2_.

In addition to CO_2_ and light, underwater *P_N_* of *T. distichum* was also strongly affected by the environmental temperature. In the temperature range tested, i.e., 10 to 35°C, there was a strong positive relationship between temperature and underwater *P_N_* until the temperature optimum was reached at 25°C after which *P_N_* declined with increasing temperature (**Figure 2D**). This is a common response of underwater *P_N_* to rising temperature as also demonstrated for two tidal seagrass species, *Thalassia hemprichii* and *Enhalus acoroides* (Pedersen et al., 2016). Being tropical species, these seagrasses showed a temperature optimum for underwater *P_N_* at 33°C, i.e., 8°C above that of *T. distichum*. The *Q*_10_ of underwater *P_N_* in *T. distichum* was somewhat lower (1.8) than that of *T. hemprichii* (2.0) and *E. acoroides* (2.8) (Pedersen et al., 2016), but it nevertheless show the strong dependency of temperature for underwater *P_N_* also in *T. distichum*. This is an important point to consider when extrapolating the current laboratory findings to the field situation since the water temperature in the Yangtze River can fluctuate from 11 to 22°C during the time of the year when the trees on the banks become submerged (Yu et al., 2021).

### 4.3 Responses of *Taxodium distichum* to long-term submergence

The trees growing on the banks of the Yangtze River can become partially or completely submerged for up to 120 days and still survive (Yang et al., 2023), and we therefore tested the response of *T. distichum* to long-term submergence. Six-month old seedlings were completely submerged for a period of 30 days with sampling of leaf tissue during the period at discrete time points. Leaf gas films quickly diminished, and the decline in gas film thickness was accompanied by a decline in underwater *P_N_* (**Figure 4C**). The strong relationship between gas film thickness and underwater *P_N_* during long-term submergence has previously been observed in rice (Winkel et al., 2014), but our study represents the first to demonstrate this relationship for a tree species.

The gas films forming on the surfaces of the submerged needles of *T. distichum* persist longer than in other species tested with hydrophobic cuticles. In the present study, the gas films were detectable up to 24 days of submerged after which they had totally vanished (**Figure 4B**). This is longer than observed for rice, which represents the only other species with superhydrophobic leaves, where gas film thickness have been followed during a submergence event. Here, it was found that gas film thickness was below the detection limit already after 7 days of submergence (Winkel et al., 2014). In *T. distichum*, the needles maintained their ability to photosynthesize also after the gas films were lost at a rate of approximately 1 µmol O_2_ m^−2^ s^−1^, which is similar to the photosynthetic rate of rice and wheat once the leaf gas films of these species have also disappeared due to long-term submergence (Winkel et al., 2014; Konnerup et al., 2017). It is still not fully understood why the loss in hydrophobicity occurs during submergence. However, the present study as well as one on wheat (Konnerup et al., 2017) clearly demonstrated that a biofilm was established during submergence (**Figure 3I**), but if this biofilm is the result or the cause of loss of hydrophobicity remains unknown.

Distinct anatomical and biochemical changes occurred during the long-term submergence event. The decline in total chlorophylls was significant with initial values at 2.1 mg g^−1^ DM and only 1.0 mg g^−1^ DM after 30 days of submergence (**Figure 5B**). While this decline will have significant consequences for the light capturing capabilities, the decline in submerged rice was even more pronounced where the initial levels are at approximately 18 mg g^−1^ DM to less than 5 mg g^−1^ DM in only 2 weeks. The decrease in chlorophyll concentration observed in the present study may be one of the important reasons for the decrease in underwater *P_N_*, as it has been shown that underwater *P_N_* of rice is positively correlated with leaf chlorophyll concentration (Winkel et al., 2014). The significant decrease in chlorophyll concentration is likely a result of palisade tissue loss in needles (**Figures 5D, E**). To our knowledge, similar observations are missing in the literature as previous studies have focused on anatomy of leaves formed during the submergence event (Mommer et al., 2007) and not on acclimation of already existing leaves. The lysis of palisade tissues observed towards the end of the 30-day submergence event might be accompanied by water infiltration in the newly formed cavities, and such water-filled cavities would slow down intra-tissue diffusion of O_2_ and CO_2_ (Armstrong, 1980). Interestingly, the parallel decline in chlorophyll concentration and leaf gas film thickness makes it difficult to identify the primary causal effect of the observed decline in underwater *P_N_*.

## 5 Conclusions and perspectives

During the 30-day submergence period, no growth or formation new leaves were observed, and therefore, *T. distichum* shows a quiescence response to submergence (cf. Bailey-Serres and Voesenek, 2008). The hydrophobicity of the needles declined during the submergence event, resulting in loss of gas films. However, the chlorophyll concentration of the needles also declined significantly, and it was therefore not possible to identify the main cause of the corresponding significant decline in underwater *P_N_*.

Several questions still remain unresolved in order to fully understand the striking ability of *T. distichum* to withstand partial or complete submergence for months. We propose that future research concentrate on unraveling the finer details in needle anatomy and biochemistry as these changes occur during submergence. For example, the lysis of palisade tissues should be further studied in order to understand if the lysis is merely a consequence of senescence processes or if the lysis is actively controlled via programmed cell death with the aim of acclimating the leaves to a low-light environment and the slow diffusion of gases in water. We also suggest to investigate if changes in cuticle structure take place beyond those involved in surface hydrophobicity. A thinning of the cuticle would greatly enhance diffusion of O_2_ and CO_2_ from the floodwater to the needles’ tissues and thereby enhance the supply of O_2_ for dark respiration or CO_2_ for underwater photosynthesis. In addition, whether the bacteria colonized on leaf surface will cause negative impacts on the leaf cells and hence affect hydrophobicity and photosynthesis, and the difference in underwater *P_N_* capacity and detailed submergence tolerance mechanisms of *T. distichum* seedlings and big trees are also interesting questions that are worthy of further investigation.

## Data availability statement

The original contributions presented in the study are included in the article/**Supplementary Material**. Further inquiries can be directed to the corresponding authors.

## Author contributions

JG: Writing – original draft, Visualization, Software, Data curation. JX: Methodology, Writing – review & editing, Validation, Supervision. YY: Writing – review & editing, Supervision, Resources. OP: Writing – original draft, Methodology, Funding acquisition, Data curation. JH: Writing – review & editing, Methodology, Funding acquisition, Conceptualization.
