# Supplementary material for: Response of underwater photosynthesis to light, CO2, temperature, and submergence time of Taxodium distichum, a flood-tolerant tree
Source: Front Plant Sci. 2024 Mar 19;15:1355729. doi: 10.3389/fpls.2024.1355729 (PMC10985249; doi:10.3389/fpls.2024.1355729)
Supplement: Supplementary file 1 [file DataSheet_1.doc]

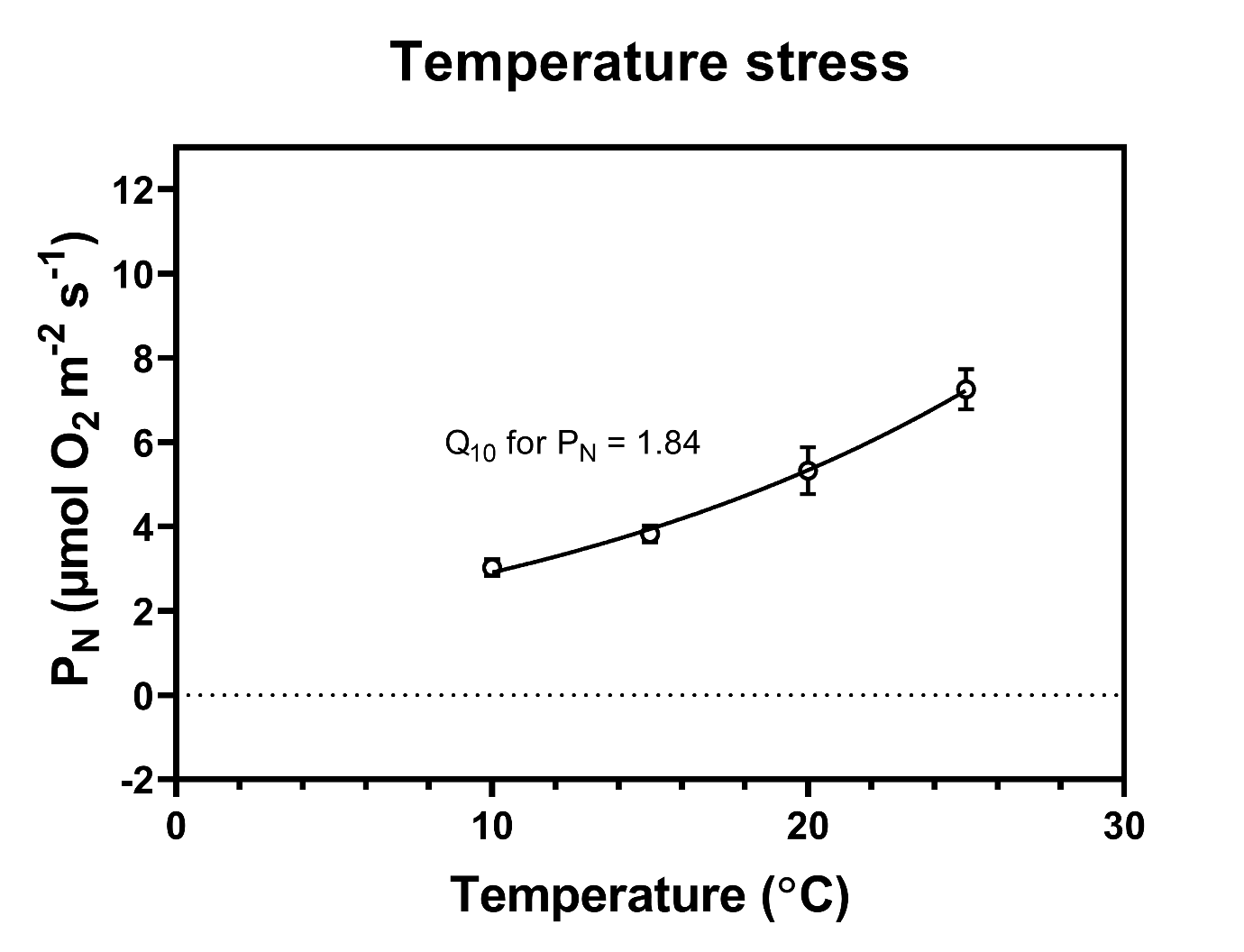


**Figure S1. Response of underwater net photosynthesis (P_N_) to temperature by *Taxodium* *distichum*.** The figure shows the response in PN in the temperature interval between 10 and 25 °C before P_N_ started to decline due to heat stress (Fig. 2D). Q_10_ was estimated using a standard exponential function fitted to the data points. Data points show the mean ± SD (n = 4)
